# Curd-Peptide Based Novel Hydrogel Inhibits Biofilm Formation, Quorum Sensing, Swimming Mortility of Multi-Antibiotic Resistant Clinical Isolates and Accelerates Wound Healing Activity

**DOI:** 10.3389/fmicb.2019.00951

**Published:** 2019-05-14

**Authors:** Sounik Manna, Ananta K. Ghosh, Santi M. Mandal

**Affiliations:** Central Research Facility, Department of Biotechnology, Indian Institute of Technology Kharagpur, Kharagpur, India

**Keywords:** antibiofilm, antimicrobial, curd, hydrogel, wound healing

## Abstract

The search for a bioactive natural antibacterial agent with wound healing properties is a common practice for the development of new-generation molecules. Antimicrobial peptides are a good alternative to antibiotics and easy-to-form hydrogels under self-assembled conditions without pH adjustment. With this in mind, the peptide pool was extracted from a formulated curd composed of a blend of probiotic bacteria such as *Streptococcus thermophilus*, *Lactobacillus casei*, and *Bifidobacterium bifidum* at an optimized ratio of 7:1:2. The water content of curd was collected by the drainage column, centrifuged, filtered through a 0.45-μM filter, and used for hydrogel preparation. Matrix-assisted laser desorption/ionization time of flight (MALDI-TOF) mass spectrometry (MS) analysis confirmed the presence of peptide pool in the extracted water. The prepared hydrogel was freeze dried, and its effect on biofilm formation, swarming mortality, antimicrobials, wound healing, and biocompatibility was subsequently verified. Transmission electron microscope (TEM) and scanning electron microscope (SEM) images revealed the fibrous network of peptides after self-assembly with non-polar n-hexane solvent and a porous structure after drying, respectively. The observed biocompatibility, antimicrobial activity, and strong wound healing activity of the developed curd-based hydrogel have opened a new platform for antibacterial ointment formulation.

## Introduction

Nowadays, wound repair is a major health concern because of its association with antibiotic-resistant bacteria. The increasing failure of conventional antibiotics to repair wounds calls for the development of new antimicrobial agents with novel targets against antibiotic-resistant microbial pathogens. In this context, the search for bioactive substances from natural resources is a common practice for the development of new agents. Antimicrobial peptides of plant and animal origin are an excellent source to search for a new antibacterial wound dressing agent (Pfalzgraff et al., 2018). Hydrogel is useful as a base material for ointment formulation and preparation of wound dressing material (Mahata et al., 2017). Hydrogels are three-dimensional, insoluble hydrophilic polymer networks capable of absorbing large amounts of water or biological fluids. Hydrogels are formed by chemical cross-linking processes such as disulfide bond formation, polymerization, or the reaction between thiols and acrylates or sulfonic acids that undergo volume changes during the transition from a sol to a gel state (Qinyuan et al., 2017). Physically cross-linked hydrogels do not undergo significant volume changes during the sol-gel transition, because they are prepared *via* the self-assembly of polymers by changing pH and temperature (Schoener et al., 2013). Often, physical cross-linking leads to the formation of weaker gels compared to chemical cross-linking, and these gels are therefore more susceptible to mechanical forces like shear stress. This can also be used to make physical hydrogels suitable as injectable materials (Qinyuan et al., 2017). Another advantage of physical cross-linking is that it mostly does not depend on the addition of organic solvents or cross-linking reagents, but offers the possibility of using these hydrogels in various biomedical applications such as in the controlled delivery of drugs in tissue engineering. Different types of bases (hydrocarbon, water-removable, and water-soluble) with antimicrobial preservatives and various surface active agents are used in the formulation of therapeutic ointments (Goodman et al., 2002). A self-assembly-based approach is also used for nanohydrogel base preparation to enhance benefits such as antimicrobial, antibiofilm, and antioxidant activities (Mahata and Mandal, 2018).

Biofilm formation by bacteria is an emerging threat in infection control as it is easily formed over hydrophobic body surfaces. Biofilm-forming bacterial cells are approximately 103 times more resistant than planktonic cells and decrease the efficacy of conventional drugs (Roberta et al., 2012). Several cationic host defense peptides including lactoferrin of milk are reported as effective antibiofilm agents (Fuente-Núñez et al., 2012; Sengupta et al., 2012). However, very few studies are done to assess the effect of ointment bases or hydrogel prepared with antimicrobial peptides and biofilm formation.

Here, we report the extraction of peptides from the drainage water from fermented curd, the preparation of hydrogel using peptide, and the assessment of its antimicrobial activity to enhance preventive measures for human health. The developed hydrogel effectively retards bacterial growth, inhibits quorum sensing (QS) and biofilm formation, and accelerates wound healing.

## Materials and Methods

Three bacterial strains, *Streptococcus thermophilus*, *Lactobacillus casei*, and *Bifidobacterium bifidum*, were obtained from the National Collection of Dairy Cultures, NDRI, Karnal, India. The cultures volume was selected on the basis of specific requirements of the product. Keeping the basic aim in mind, the curd starter was formulated, which would give a firm body and consistency in texture, thickness, color, flavor, taste, acidity, and shelf life.

### Preparation of Curd

Strains were freshly subcultured in MRS (de Man, Rogosa, and Sharpe) agar medium and incubated at 37°C for 14 h. Different volumes of log phase grown cultures (OD at 600 nm about 0.5) were used to prepare curd. The higher inoculum volume of *S. thermophilus*(70–80%) was used, while the other two cultures (*L. casei* and *B. bifidium*) were used at 10% and 20%, respectively. After using various combinations and ratios of cultures, the best combination and optimized ratio of 7:2:1 (for *S. thermophilus*, *L. casei*, and *B. bifidum*, respectively) were obtained.

### Peptide Isolation

An acid-methanolic extract of curd water was prepared following the method of Nayak et al. (2018). In brief, curd-drained water was collected and then diluted 10 times with a solution of methanol/glacial acetic acid/water (90:1:9), mixed thoroughly, kept at room temperature for 10 min, and then centrifuged at 12,000 rpm for 30 min at 4°C. The supernatant was collected, the methanol was evaporated through a rotary evaporator. To remove the lipid content, the same volume of n-hexane was added, and centrifuged at 12,000 rpm for 10 min at 4°C. The upper fraction containing lipid was removed, and the lower polar fraction containing peptides was freeze-dried and dissolved in 0.1% trifluoroacetic acid (TFA).

Further purification of curd peptides was achieved through reverse-phase high performance liquid chromatography (HPLC) (HPLC, Agilent 1100 series, Agilent Technologies, United States) by using a semipreparative C18 column (Hypersil BDS-C18, 5 mm, 4.5 mm × 250 mm). The solvent system used was acetonitrile containing 0.05% TFA (A) and 0.05% TFA (B) in water. The gradient of solvent B was used as described earlier (Mandal et al., 2013). The elution from the column was monitored at 215 nm in a diode array detector with a flow rate of 1 ml min^-1^. Different peak fractions were collected and lyophilized to dryness.

### Hydrogel Preparation

The lyophilized peptide pool was dissolved in 0.1% aqueous TFA at a final concentration of 10 μg ml^-1^. To this, zinc nitrate solution was added to a final concentration of 50 mg ml^-1^ and allowed to form hydrogel. Concentration of the zinc nitrate solution was optimized for the formation of proper hydrogel.

### Characterization of Hydrogel

Total peptide pool was characterized using a matrix-assisted laser desorption/ionization time of flight (MALDI-TOF) mass spectrometer. The purified lyophilized peptide pool was resuspended in methanol and 4 μl of peptide was mixed with 4 μl of matrix (CHCA, 10 mg/ml). The mixture (1 μl) was spotted onto the MALDI 100-well stainless-steel sample plate and allowed to air dry prior to analysis (Mandal et al., 2009). The spectra were recorded in positive ion linear mode. For reproducible result of the spectrum, the sample was separately spotted thrice and analyzed in a MALDI-TOF mass spectrometer (Mandal et al., 2013).

For Fourier transform infrared (FTIR) analysis, the curd peptide-based hydrogel is placed onto a KBr pellet and then dried completely. The spectra of the dried specimens were recorded on a Shimadzu 8400 FTIR spectrophotometer. Absorbance spectra were recorded from 4,000 to 400 nm with a 4 cm^-1^ resolution, after subtracting absorbance background (Mandal et al., 2017).

For SEM images of the self-assembled curd-peptide-based hydrogel, 5 ml of stock solution (10 mg ml^-1^) was placed on the glass coverslip and dried under room temperature. Samples were fixed onto a graphite stub and kept in an auto sputter coater (E5200, Bio-Rad, Hadapsar, Pune, India) under low vacuum for gold coating up to 120 s. Surface morphology was analyzed by a scanning electron microscope (JEOL JSM 5800, GenTech Scientific, NY, United States) operated with an accelerated voltage between 5 and 20 kV (Mandal et al., 2017).

### Antimicrobial Assay

The lyophilized peptide pool was dissolved in 0.1% aqueous TFA and lyophilized hydrogel was added in sterile distilled water to test its antimicrobial activity against antibiotic-resistant *S. aureus* and *P. aeruginosa* strains following the protocol described by Wang et al. (2018). The strains were described earlier as used in this study (Samanta et al., 2013). Minimum inhibitory concentration (MIC) was determined at the lowest concentration of compound required to inhibit the growth of the test strain without showing any turbidity (Nayak et al., 2018).

### Flow Cytometry Analysis

Flow cytometry analysis was performed following the protocol of Roymahapatra et al. (2015). Total number of dead cell population was determined by dot plot analysis in Cell Quest Pro software attached to a fluorescence activated cell sorter (Becton Dickinson India).

### Biofilm Quantification

Both crystal violet (CV) staining and XTT (2,3-bis-(2-methoxy-4-nitro-5-sulfophenyl)-2H-tetrazolium-5-carboxanilide) reduction assay (Ramage et al., 2001) were performed to quantify the biofilm formation as described by Mandal et al. (2013).

### Fluorescence Microscopic Analysis

Biofilm-containing bacteria were stained with Syto-9 following the supplier’s protocol (Invitrogen, Thermo Fisher Scientific, India) and washed thrice with PBS (1×) to remove the debris. Images of stained bacteria were captured with an inverted fluorescence microscope (Olympus IX 70 fluorescence microscopy).

### Swimming Motility Assay

Swimming motility assay was performed in 0.3% LB medium following the method described by Fuente-Núñez et al. (2012). One-microliter aliquots of mid-log-phase (OD_600_ of 0.45–0.55) cultures was inoculated and mixed with increasing concentrations of hydrogel. The diameters of the swimming zones were measured after incubation for 15 h at 37°C. Swimming motility was determined by measuring the diameters of the twitching zones. All assays were performed in triplicate.

### Inhibitory Effect of Hydrogel on Quorum-Sensing Activity of *Chromobacterium violaceum* MTCC 2656

The effect of hydrogel on the production of QS-mediated violacein in *C. violaceum* was studied by the agar well assay. *C. violaceum* was mixed with molten nutrient agar of and then poured into plates. A well of 5-mm diameter was made at the center of the agar plates; 100 ml of hydrogel was added and incubated at 37°C for 16 h. The appearance of clear zone of inhibition of bacterial growth surrounding the well confirmed the inhibition of QS (Ghosh et al., 2014).

### Determination of Quorum Sensing-Inhibitory Concentration of Hydrogel With Reference to Violacein Production

One hundred microliter of log phase cells of grown *C. violaceum* bacteria MTCC 2656 (2.5 × 10^6^ CFU ml^-1^) were used to inoculate 10 ml of LB medium containing different concentrations of peptide-derived hydrogel (100–1,000 μg ml^-1^) and incubated at 37°C for 16 h with shaking. The production of the violacein pigment was quantified following the method of Ghosh et al. (2014). In brief, 1-ml culture was centrifuged at 13,000 rpm for 3 min, and the pellet was completely dissolved in 1 ml of DMSO by vigorously vortexing and centrifuged at 10,000 rpm for 10 min to remove the cells. The absorbance of the supernatant was measured at 585 nm in a digital spectrophotometer (Thermo Spectronic UV 1). The maximum OD_585_ value observed for control cells without any hydrogel was considered as 100% violacein production. The percentage (%) inhibition in violacein production was calculated as follows: % inhibition in violacein production=[(OD_585_ value observed in the absence of hydrogel - OD_585_ observed in the presence of a defined quantity of hydrogel)/OD_585_ value observed in the absence of hydrogel] × 100.

### Scratch Assay for Wound Healing Activity

Human keratinocyte cells (HaCaT) were cultured on lysine-coated coverslips (18 mm × 18 mm) in 35-mm petri dishes in DMEM-F12 medium at a 37°C incubator at 5% CO_2_ atmosphere. An *in vitro* scratch wound was made by using a 200-μl sterile pipette tip on confluent HaCaT population attached to the coverslip. Plates containing coverslips were washed twice with PBS (pH 7.4). The curd peptide hydrogel was prepared in DMEM-F12 complete media and added to a plate with a final concentration of 10 μg/ml. The coverslip without hydrogel was used as the control. At different time points (0, 8, and 16 h), the wound pictures were observed under a phase contrast microscope (10×, Zeiss Axio Observer Z1, Carl Zeiss, Germany) and photographed. The wound width was measured by Axiovision software (Version 4.7.2, Carl Zeiss, Germany) to calculate fraction of wound healing (Fronza et al., 2009).

## Results

### Curd Formation and Hydrogel Preparation

**FIGURE 1:**
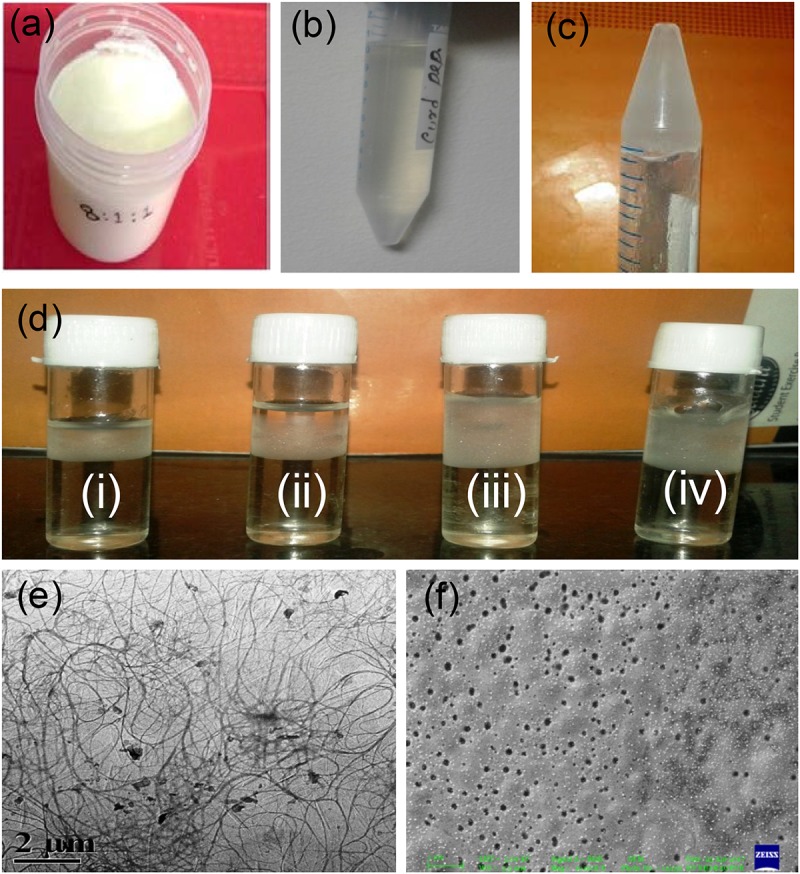
Hydrogel preparation from extracted curd peptides. Prepared curd in the laboratory **(a)**; extracted curd peptide pool **(b)**; hydrogel from peptide **(c)**; optimization of Zn(NO_3_)_2_ ion **(i)**, 25 μg ml^-1^; **(ii)**, 50 μg ml^-1^; **(iii)**, 75 μg ml^-1^; **(iv)**, 100 μg ml^-1^ for hydrogel preparation **(d)**; TEM image of self-assembled curd peptide **(e)** and TEM image of hydrogel **(f)**.

Formation of different amounts of whey on the top of the prepared curd of different textures using different amounts of three types of probiotics was observed. The curd with the most firm body and the best textural properties was obtained when probiotics *S. thermophilus*, *L. casei*, and *B. bifidum* were used at a ratio of 7:1:2 after optimization (data not shown here) (Figure 1a). Peptides were isolated from curd-drained water (Figure 1b,c). Figure 1d shows the effect of different concentrations of zinc nitrate for the formation of hydrogel. The optimum concentration of zinc nitrate that helps to prepare hydrogel was determined as 100 mg/ml.

### Morphological Characterization of Hydrogel

The morphology of the hydrogel prepared was observed through FE-SEM and transmission electron microscope (TEM). The TEM micrograph (Figure 1e) shows their cross-linked pattern with a large proportion of self-assembled curd peptides along with zinc ion. Interestingly, the images from a low dilution of colloidal solution revealed a uniform nanoparticle-like structure that may appear after the self-assembly of the individual peptide. The SEM image of the gel showed a uniform porous shell-like structure (Figure 1f). This may be due to the assembly or organization of the cross-linked structure between the peptides of curd with zinc ions. Peptides have a strong non-covalent interaction between them, which forms a porous hydrogel structure. The strong physical cross-linking *via* hydrogen-bonding interaction between the multiarmed peptide chain and the zinc ion assisted in the formation of a well-organized self-assembled hydrogel.

### HPLC and MALDI-TOF Analysis of Curd Peptides

**FIGURE 2:**
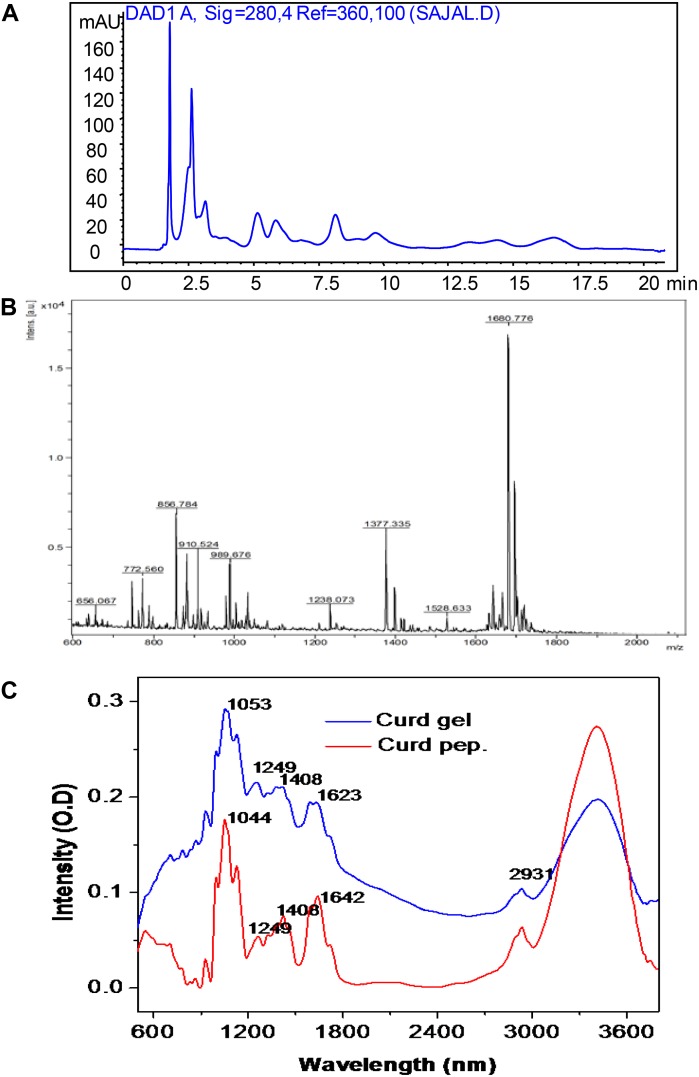
High-performance liquid chromatographic profile of extracted curd peptide **(A)**; matrix-assisted laser desorption/ionization time of flight (MALDI-TOF) mass spectrometric profile of extracted curd peptide **(B)**; Fourier transform infrared (FTIR) spectroscopic overlay profile of extracted peptide pool (red) and prepared hydrogel (blue) **(C)**.

The methanol and acetic acid extracted curd peptides were separated through reverse-phase HPLC. The HPLC chromatogram revealed the presence of peptide peaks in the curd extracts (Figure 2A). The curd-derived peptide pool was also analyzed through MALDI-TOF mass spectrometry (MS) (Figure 2B) in positive ion linear mode and revealed the presence of nine peaks. The ions were obtained with m/z 656.06, 772.56, 856.78, 910.52, 989.67, 1238.07, 1377.33, 1528.63, and 1680.77.

### FTIR Analysis of Curd Peptides and Gel

The FTIR spectra were obtained from both extracted peptides of raw curd and hydrogel prepared from these peptide-derived curd gels. In both cases, a major broad peak was observed at bands from 3,400 to 3,600 cm^-1^. In this region, the peaks correspond to the OH and COOH stretching. The peak at 2,931 cm^-1^ corresponding to –CH– stretching was observed in the same region for both cases and confirms that the CH bond is not involved in gel formation. Interestingly, the peak observed at 1,623 cm^-1^ for curd hydrogel was found at 1,642 cm^-1^ for peptide fraction (corresponding to the NH out of plane, and in some cases, it is also responsible for -C-O- stretching). Therefore, it seems that in this region, the major shift occurs due to the involvement of an amide (–RCONH–) bond. Another major shift occurs at a peak of 1,044–1,053 cm^-1^, which is mainly responsible for the –C–O– stretching in the primary alcohol structure. It is clear from the FTIR analysis that the gel formation occurred due to the vibration change at –C=O– and –RCONH– in peptides, and H-bonds helped to create the hydrogel after cross-linking with each other (Mansur et al., 2008; Figure 2C).

### Antimicrobial Activity

**FIGURE 3:**
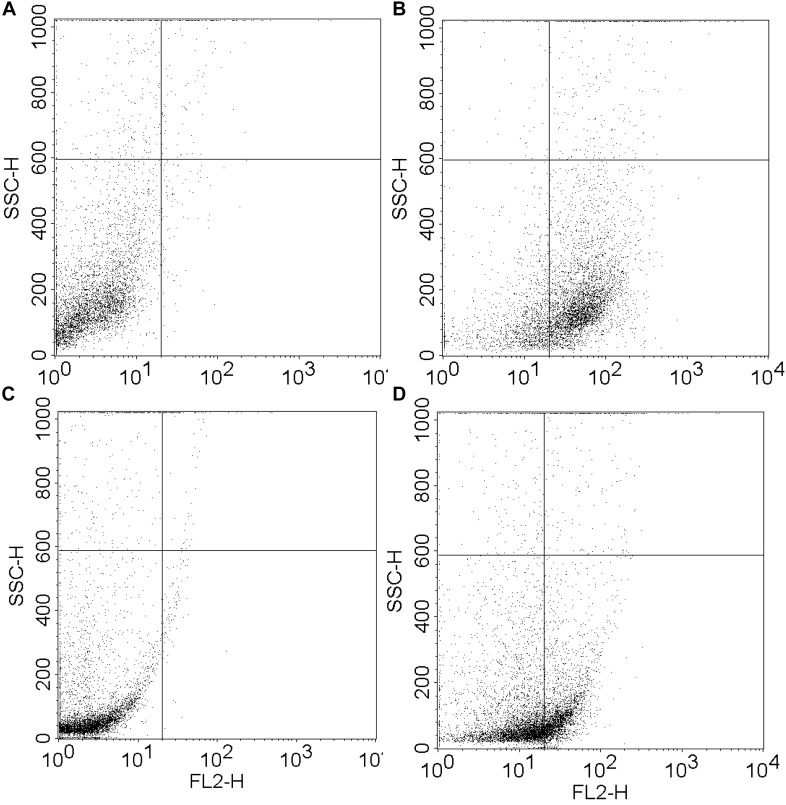
Fluorescence activated cell sorter (FACS) analysis of hydrogel-treated *S. aureus* and *P. aeruginosa* at a concentration of 16 μg ml^-1^. Dead cell percentages were calculated as 62.54% for *S. aureus* **(A,B)** and 34.88% for *P. aeruginosa* **(C,D)**.

**FIGURE 4:**
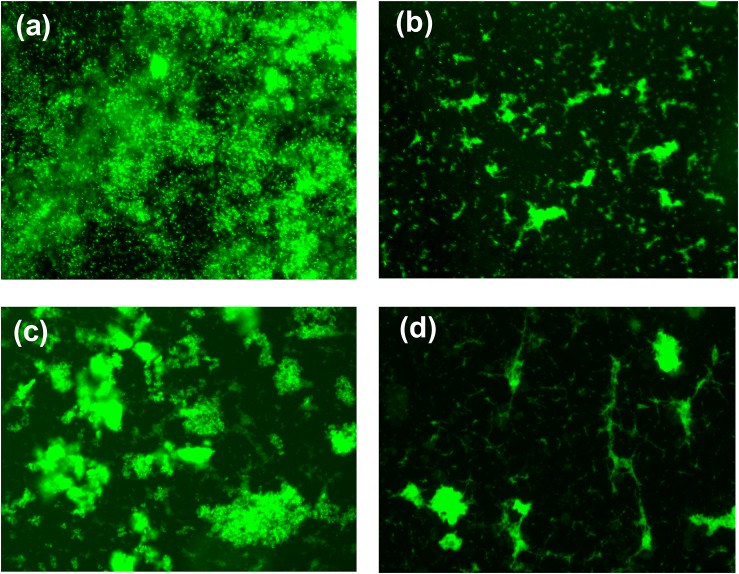
Fluorescence microscopic image of bacterial biofilm formation untreated *S. aureus* **(a)**, hydrogel-treated *S. aureus* **(b)**, untreated *P. aeruginosa* **(c)**, and hydrogel-treated *P. aeruginosa* **(d)**. Biofilm was stained with SYTO 9 before capturing the image.

**FIGURE 5:**
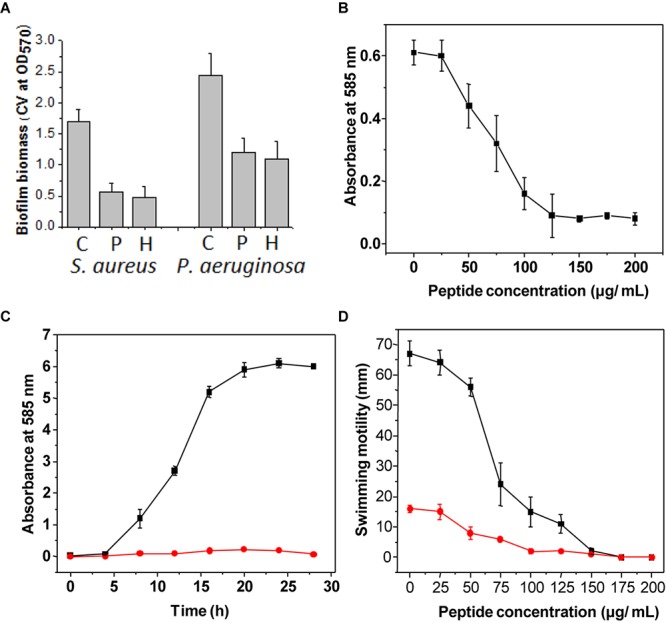
Assay of biofilm formation after staining with crystal violet stain **(A)**. Bar graph indicates the biofilm formation of *S. aureus* and *P. aeruginosa* treated with only curd **(C)**, biofilm formation when treated with extracted peptide (P), and treated with hydrogel (H). The concentration used was 32 μg ml^-1^. Violacein production by the strain *C. violaceum* treated with different concentrations of peptide **(B)**. Violacein production by the strain *C. violaceum* in batch culture grown with (red line) and without (black line) peptide **(C)**. Assay of swarming motility was evaluated on LB agar plates treated with different concentrations of hydrogel **(D)**. The diameters (in cm) of the swim zones were measured after incubation for 20 h at 37°C. The swim motility was studied in medium supplemented with different concentrations (0–200 μg ml^-1^) of hydrogel against both strains, with *S. aureus* indicated by the red line and *P. aeruginosa* indicated by the black line. All experiments were done in triplicate. Data are the mean of triplicates ± SE.

The MIC of both peptide and hydrogel was evaluated against *S. aureus* and *P. aeruginosa* and it was found to be more active against *S. aureus* (MIC = 32 μg/ml) in comparison to *P. aeruginosa* (MIC = 64 μg/ml; Table 1). Flow cytometry analysis of hydrogel treatment at a concentration of 16 μg/ml showed a significant percentage of dead cells for *S. aureus* (62.54%) and *P. aeruginosa* (34.88%) (Figure 3). Analysis of biofilm formation was visualized with fluorescence microscope image analysis (Figure 4) using SYTO 9 stain revealed a significant reduction of biofilm formation (Figure 4) by the bacteria treated with curd peptide-derived hydrogels. Biofilm formation was quantified using crystal violet (CV) stain as shown in Figure 5A.

**Table 1:** Minimum inhibitory concentration (MIC) of peptides, peptide-derived hydrogel, and a few antibiotics.

Antibacterial agents	Minimum inhibitory concentration (μg ml^-1^)	
	*S. aureus*	*P. aeruginosa*
Curd extracted peptides	128	524
Peptide-derived hydrogel	32	64
Ceftazidime	31.25	31.25
Vancomycin	15.62	31.25
Piperacillin	31.25	62.50
Ofloxacin	128	128


### Determination of Growth and Violacein Production of *C. violaceum* MTCC 2656

Violacein production from *C. violaceum* was monitored after incubation with different concentrations of peptide hydrogel, and a decrease in violacein production was observed with the increase of hydrogel in a dose-dependent manner. Hydrogel at a concentration of half of the MIC value completely stopped the violacein production (Figure 5B,C).

### Inhibition of Swimming Motility

Swimming motility is an important criterion for biofilm formation and is regulated by flagellar movement of bacteria. Swimming motility was tested by incubating *S. aureus* and *P. aeruginosa* with peptide-derived hydrogel, and it was observed that the motility rate of both bacteria was significantly reduced after treatment (Figure 5D).

### Wound Healing Assay

**FIGURE 6:**
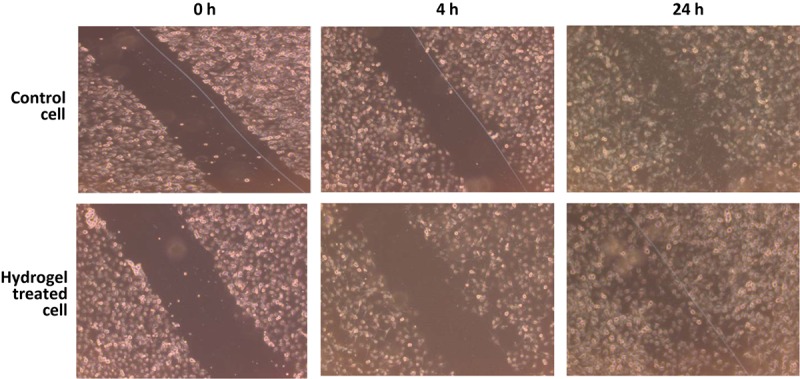
Wound-healing assay was performed to examine the migration of human keratinocyte (HaCaT) cells through scratch assay. Photographs were taken at 0, 4, and 24 h following the initial scratch.

Wound healing activities were performed against immortalized human keratinocyte (HaCaT) cell lines. HaCaT cells play a crucial role in regulation of skin epidermal tissue regeneration and were used as a model cell line to test *in vitro* wound healing activity. The survival rate of HaCaT cells was evaluated under different concentrations of hydrogel. Proliferation of most of the cells occurred at a hydrogel concentration of 0.5 μg/ml. Therefore, a hydrogel concentration of 0.5–1 μg/ml was selected for the wound healing (scratch) assay, and it was observed that maximum wound healing occurred after 24 h of incubation in comparison to control. These results from *in vitro* scratch assay with hydrogel indicate that this hydrogel has the potency to be used as a wound healing agent (Figure 6).

## Discussion

Milk and milk products are used as essential food for human nutrition (Goodman et al., 2002; Laakkonen and Pukkala, 2008) because these are a good source of protein, calcium, vitamins, and trace elements like zinc (Jelen, 2005; Miller et al., 2007). They have a significant effect against numerous diseases and they boost the immune system to fight against pathogens. In milk and fermented milk products, different types of proteins and peptides are also present, which mostly act against cancer cells (Tsuda et al., 2000). In addition, several bioactive peptides in milk-derived raw or fermented products are well documented as regards to their role against various pathogens and their potential in biotechnological applications (Mandal et al., 2014; Khan et al., 2018; Fideler et al., 2019).

Hydrogel-based dressing materials provide hydration to wounds and accelerate the healing process for faster pain relief. Different base materials are reported to be used for hydrogel preparation (Liu et al., 2018). Here, a curd-extracted peptide pool is used as the base material for the formulation of hydrogel. The presence of different peptides with various molecular weights has been confirmed by HPLC and MALDI-TOF MS analysis. The prepared hydrogel formed from these curd peptides is found porous in nature. A TEM image revealed that the rigid fibrous structure built *via* cross-linking may provide the possible cause of hydrogel formation. Earlier, it has been reported that zinc ions are able to trigger the peptide-based hydrogel formation (Tao et al., 2018). After a detailed mechanistic analysis, it was also stated that the network of weak non-covalent interactions, such as π–π interactions, hydrogen bonding, and van der Waals forces, is capable of forming nanofibers. The carbonyl groups of peptides may perhaps chelate the zinc ions and accelerate the gelation process. Here, we have also observed a significant vibrational change at –C = O- and –RCONH– in peptides. The presence of cationic metal (Zn) ions helps in self-assembly formation and supramolecular gel formation. The cross-linking that occurred here may be due to the coordination of the carboxylic acid group of peptides to the Zn ions. Earlier, several metal ions such as Fe(II), Ni(II), Cu(II), and Zn(II) were used to transform the copolymer to gel phase (Kotova et al., 2015).

The healing process depends on several factors such as epithelization, contraction, connective tissue deposition, regeneration of new collagens, and inflammatory responses (Anderson and Hamm, 2014). It has also been reported that oxidative stress from neutrophile-derived oxidants and myeloperoxidase activity trigger chronic tissue damage and accelerate wound formation. Therefore, reactive oxygen species (ROS) play a crucial role in chronic wound inflammation, and scavenging of ROS developed by bioactive copolymer may be an option to make the healing process faster. When bacterial infection occurs, then the ROS production increases because of the activation of NADPH oxidase (Chen et al., 2013). The developed material shows both antibacterial and strong antibiofilm activity to prevent the colonization of bacteria in wounds and subsequently to promote the healing process. Interestingly, the hydrogel has significantly reduced swimming motility and, subsequently, biofilm formation by decreasing flagellar movement. Earlier, it has been reported that several chemotaxis genes are upregulated whereas the genes related to flagellar movement are downregulated (Sarah and Daniel, 2013). Bacterial behavior within biofilms is controlled by the QS signaling. The disruption of biofilm is necessary to effectively control the pathogens by antibiotics. These quorum-sensing inhibitors can competitively inhibit the QS signaling system (Drenkard, 2003). Here, a milk-peptide-derived hydrogel is shown to inhibit QS. Quorum-sensing inhibitors have the potential to control bacterial infections without disturbing the bacterial cells and to subsequently minimize the role of antibody development (Zeng et al., 2008).

## Conclusion

A unique probiotics-based curd preparation protocol has been developed. A cationic peptide pool is extracted from the developed curd and transformed into hydrogel after self-assembly of peptide and subsequent cross-linking with zinc ions. The hydrogel showed antibacterial and antibiofilm properties, reduction in swarming motility of pathogenic bacteria, inhibition of QS, and good wound healing activities. These results suggest that milk-peptide-based hydrogel may be used for skin tissue regeneration and repair during injury. The remaining curd after extraction of water may facilitate the growth of probiotics in the human gastrointestinal tract.

## Author Contributions

SM performed the experiments. AG helped to write the manuscript and critically analyzed the experimental data. SMM conceived the idea and wrote the manuscript.

## Conflict of Interest Statement

The authors declare that the research was conducted in the absence of any commercial or financial relationships that could be construed as a potential conflict of interest.
